# Methylene blue associated retinal toxicity

**DOI:** 10.1186/s40942-025-00760-8

**Published:** 2025-12-04

**Authors:** Sidra Zafar, Martin Calotti, Timothy T. Xu, Justin Muste, Theodore Bowe, Luis Acaba-Berrocal, Brian Cheng, Samir N. Patel, Yoshihiro Yonekawa, Jose S. Pulido, Jordan D. Deaner

**Affiliations:** 1 Wills Eye Hospital Retina Service, Mid Atlantic Retina, Thomas Jefferson University, 840 Walnut Street, Suite 1020, Philadelphia, PA 19107 USA; 2 The Vickie and Jack Farber Vision Research Center, Philadelphia, PA USA

## Abstract

**Background:**

To describe a case of inadvertent methylene blue use during pars plana vitrectomy (PPV) for an epiretinal membrane (ERM).

**Case:**

A 69-year-old man presented with 1 day of severe vision loss in his left eye. Two days prior to presentation, he had undergone PPV that was complicated by accidental methylene blue use. Visual acuity (VA) at presentation to our institution was CF from his baseline VA of 20/60. Optical coherence tomography (OCT) demonstrated diffuse hyperreflectivity and thickening of the inner retinal layers. The patient was started on oral prednisone with no improvement. On postoperative month 1, VA was HM. OCT showed disruption of the inner retinal architecture, inner retinal layer thinning and focal disruption of the outer retina layers superiorly.

**Conclusion:**

Methylene blue may be associated with severe retinal toxicity. Given its similarity to other vital dyes in ophthalmology, care must be taken to avoid its inadvertent administration.

In this letter, we report a case of inadvertent intravitreal methylene blue injection during an epiretinal membrane peel resulting in rapid, severe vision loss.

A 69-year-old male with no significant past medical history and a past ocular history of glaucoma was referred from an outside institution with concern for left eye retinal toxicity. Approximately 2 days prior to presentation, the patient underwent a vitrectomy in his left eye (OS) for an epiretinal membrane (ERM) that was complicated by the inadvertent intravitreal injection of methylene blue instead of intended brilliant blue. The accidental use of methylene blue was realized during surgery, after which the vitreous chamber was copiously irrigated. On postoperative day 1, the patient was noted to have a significant decrease in his visual acuity (VA) OS to hand motions (HM) from 20/60 preoperatively. He was subsequently transferred to our institution for further evaluation and consideration of intravenous steroids.

On his initial presentation to our institution (postoperative day 2), VA was 20/25 OD and counting fingers OS. Intraocular pressures (IOP) were 12 OD and 6 OS. Slit-lamp examination OD was unremarkable except for the presence of pseudophakia. Slit-lamp examination OS was similarly unremarkable with no evidence of anterior segment or anterior vitreous inflammation. Dilated fundus exam (DFE) OS showed a subtle patch of retinal whitening in the superior macula (Fig. 1A). Fluorescein angiography (FA) showed normal choroidal, arterial and venous filling. There were no obvious areas of capillary non-perfusion appreciated. However, there were multifocal areas of increasing hyperfluorescence noted along the perimacular vessels (Fig. 1B). Fundus autofluorescence (FAF) revealed a single focus of mixed hyper- and hypoautofluorescence that corresponded to one of the areas of later hyperfluorescence on angiography. There was also a mild opaqueness noted overlying the vessels which may be suggestive of loss of retinal transparency in the setting of edema (Fig. 1C). Optical coherence tomography (OCT) of the macula demonstrated diffuse hyperreflectivity and thickening of the inner retinal layers (Fig. 1D).

**Fig. 1:**
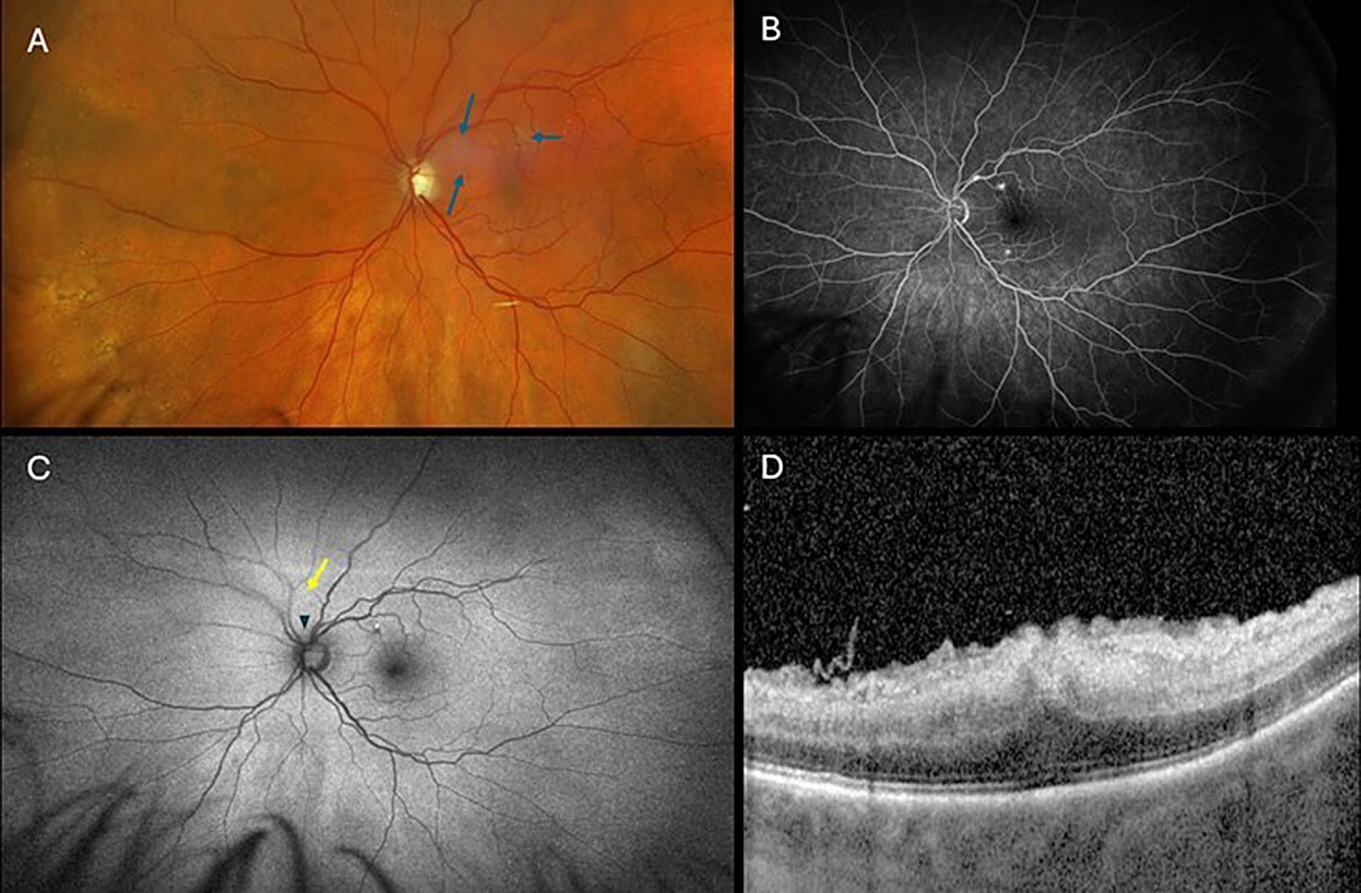
(**A**) Postoperative day 2 (POD2) ultrawide fundus image of the left eye showing a subtle area of retinal whitening superiorly as delineated by the blue arrows, (**B**) fluorescein angiogram (FA) with pinpoint areas of hyperfluorescence along the vessels, (**C**) fundus autofluorescence (FAF) with a single focus of mixed hyper- and hypoautofluorescence that corresponded to one of the areas of later hyperfluorescence on angiography and mild opaqueness overlying the superior and superonasal retinal vessels (yellow arrow). The optic nerve rim appears thin consistent with patient’s history of glaucoma (blue triangle) (**D**) Horizontal OCT centered on the fovea shows diffuse inner retinal layer hyperreflectivity and thickening

The patient was started on oral prednisone 40 mg to help reduce any secondary inflammation, with plans for weekly taper by 10 mg. On follow-up, 1 week following surgery, the patient’s examination remained unchanged, and his VA had declined to HM OS. On follow-up, 1 month after surgery, the patient’s VA OS remained HM. FAF demonstrated resolution of the opaqueness that was noted to overly the retina vessels at the earlier visit likely in the setting of the improved retinal edema and hyperreflectivity and instead demonstrated new multifocal areas of hyper-FAF that appeared deep in the retina (Fig. 2A). OCT findings were notable for disruption of the inner retinal architecture, loss of nerve fiber layer and inner retinal layer thinning. The outer retina layers appeared largely unaffected except for single focus of outer retinal disruption superiorly (Fig. 2B and C). Electroretinogram (ERG) obtained at this visit showed a normal *a*- and *b-*wave response under light-adapted conditions OS compared to OD. Under dark-adapted conditions, the amplitude of the b-waves appeared mildly decreased OS compared to OD.

In summary, methylene blue may be associated with a transient vasoconstrictive effect which leads to inner retinal ischemia and loss, while the outer retinal architecture is largely unaffected. The vision loss was profound and irreversible.

**Fig. 2:**
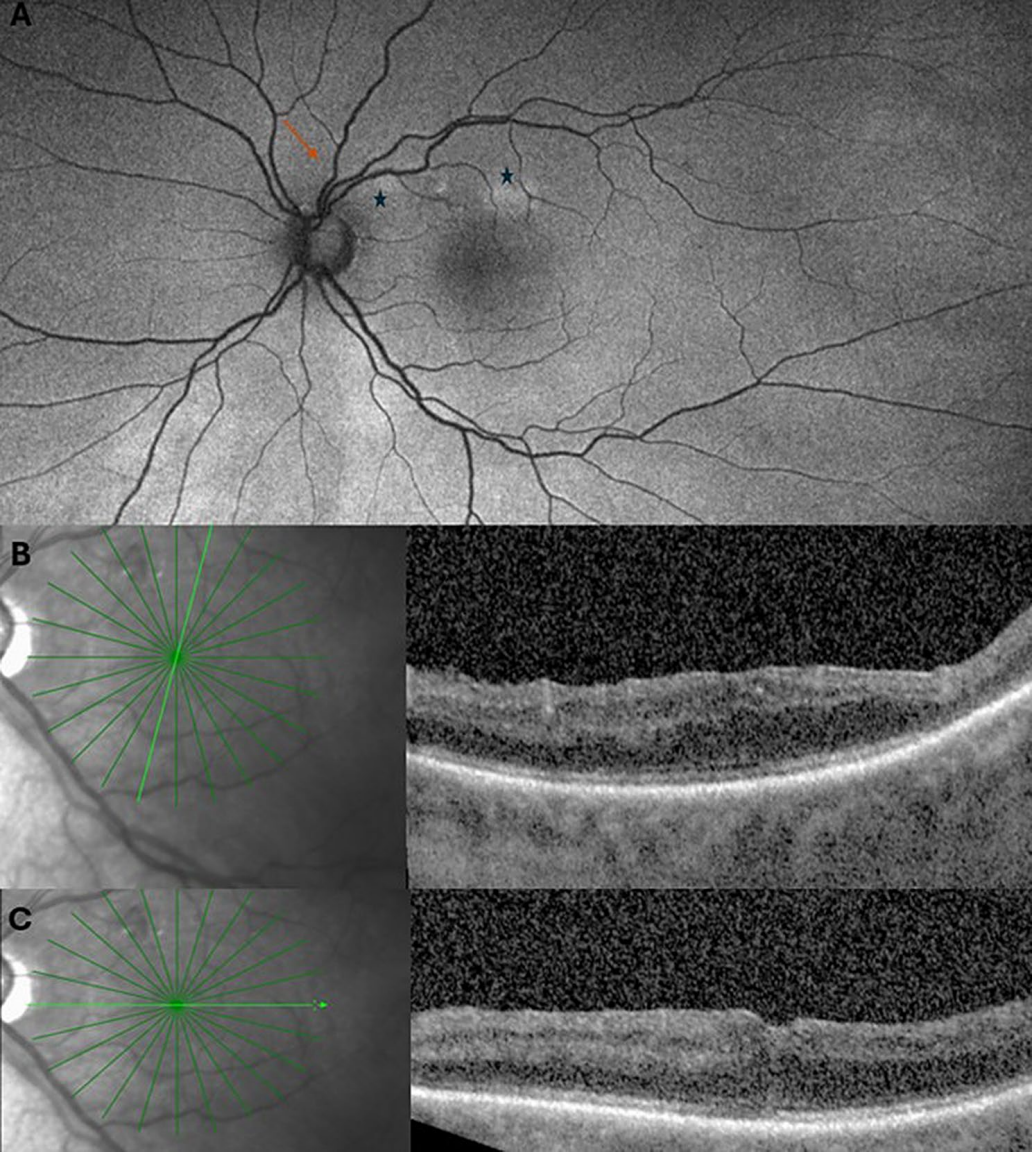
(**A**) Postoperative month 1 (POM1) fundus autofluorescence (FAF) with resolution of the previously seen opaqueness overlying the retinal vessels (orange arrow) in the setting of improving retinal hyperreflectivity and with new areas of hyper-autofluorescence (black stars). The optic nerve rim also appeared more cupped suggestive of nerve fiber layer loss (blue triangle) (**B**) POM1 OCT notable for disruption of the inner retinal architecture primarily, inner retinal layer thinning and focal disruption of the outer retinal layers and (**C**) Horizontal OCT centered on the fovea shows complete loss of the nerve fiber layer

## Data Availability

Not applicable.

